# Sublingual sufentanil tablet system Zalviso® for postoperative analgesia after knee replacement in fast track surgery: a pilot observational study

**DOI:** 10.1186/s40634-018-0123-y

**Published:** 2018-03-20

**Authors:** Marco Scardino, Tiziana D’Amato, Federica Martorelli, Giorgia Fenocchio, Vincenzo Simili, Berardo Di Matteo, Dario Bugada, Elizaveta Kon

**Affiliations:** 1 Department of Anesthesia, Humanitas Research Hospital, Via Manzoni, 56, Rozzano, Milan, Italy; 2 Center for functional and biologic reconstruction of the Knee, Humanitas Clinical and Research Institute, Via Manzoni 113, 20089 Rozzano, Italy; 3 Department of Medicine and Surgery, Parma University, Parma, Italy; 4 Department of Anesthesia, ICU and Pain Medicine, Parma University Hospital, Parma, Italy

**Keywords:** Fast track, Total knee arthroplasty, Sublingual sufentanil tablet system, Continuous femoral nerve block, Zalviso®

## Abstract

**Background:**

Currently many TKA protocols rely on multimodal analgesic protocols with patient-controlled analgesia systems that administer opioids through a patient controlled IV infusion pump, in addition to concomitant peripheral nerve blocks and local anesthetics. Although effective, PCA IV opioids do not provide optimal results with fast track rehabilitation protocols.

**Methods:**

The present is a retrospective study comparing the novel sublingual sufentanil PCA system (SSTS) to our standard of care foreseeing continuous femoral nerve block (cFNB) within a multimodal analgesic in a TKA fast-track protocol. The study evaluated 95 patients on SSTS (SSTS group) and 87 on cFNB (cFNB/control group) and collected data on numeric rating scores for pain from day 1–3 after surgery (T1, T2, T3), both at rest (NRS) and during movement (mNRS), patient’s ability to walk, need for supplementary analgesia (rescue dose), occurrence of adverse effects, length of hospital stay, and usability rating for SSTS by both patients and hospital staff.

**Results:**

NRS at rest was lower in the cFNB than in the SSTS group for all 3 days after surgery, whereas mNRS scores were lower in the SSTS group at all time points measured. Adverse effects were significantly fewer among patients of the SSTS group (6% patients) than those of the cFNB (74% patients) (*p* <  0.001). Rescue doses were needed by 5% of SSTS patients vs 60% of cFNB. The fewer adverse events and lower pain scores for the SSTS group were associated to a notably better ability to ambulate, with all patients (100%) of the SSTS group being able to stand and walk for 10 m from T1 on; patients in the cFNB group showed a slower recovery with only 40% being able to stand and walk on T1, 70% on T2 and 85% on T3. All patients of the SSTS group had a length of stay of 4 days (day of surgery plus 3 after) as foreseen by the fast track protocol, in comparison only 36% of cFNB. Lastly, patient and nursing staff judged SSTS easy to use.

**Conclusion:**

Our experience suggests that SSTS is a valuable strategy for routine postoperative analgesia following TKA in the context of a multimodal analgesic approach within the fast-track setting.

## Background

In recent years, a number of surgical procedures, including total knee arthroplasty (TKA), have turned to the fast track approach (Kehlet & Thienpont, 2013; Rodriguez-Merchan, 2015; den Hertog et al., 2012; Turnbull et al., 2017; Bugada et al., 2017), which focuses on (i) providing the patient the most suitable peri-operative conditions possible while (ii) facilitating early rehabilitation (Kehlet & Thienpont, 2013) and quickest patient recovery by reducing to the least negative stimuli or risks (i.e. urinary catheter, articular drainage, dislodgement, programming errors, thrombosis, pneumonia, anaemia, bladder infection) (Allegri et al., 2016; Schein et al., 2009; Panchal et al., 2007). Accordingly, the success of fast-track programs is largely influenced by pain management and the patient’s ability to engage in effective rehabilitation (Kehlet & Thienpont, 2013; Carli et al., 2011). Commonly used anaesthetic/analgesic strategies that are known to perform best in orthopaedic surgery may not, however, meet performance requirements for the fast track recovery (Sacerdote et al., 2016; Rawal, 2016).

This is also true for continuous femoral nerve block (cFNB), which has been acknowledged as the most effective in TKA (Chan et al., 2014; Ilfeld, 2017; Albrecht et al., 2016). In fact, cFNB features a higher or equal efficacy and prolonged relief compared to other standard techniques (epidural, local infusion anesthetic, single shot femoral nerve block, patient controlled analgesia alone) and involves a lower opioid consumption and fewer opioid-related side effects (Lotsch, 2005); yet, its effects on the sensory and motor nerves increase muscular weakness and the risk of fall (Pelt et al., 2014; Elkassabany et al., 2016; Lotsch, 2005; Mudumbai et al., 2014), suffers from the limited half-life of the local anaesthetic and relies on the use of catheter for continuous block, entailing the traditional IV-related complications (dislodgment, occlusion, infection, etc) (Allegri et al., 2016; Albrecht et al., 2016).

Similarly, traditional opioid patient-controlled analgesia (PCA), which are extensively used concomitantly with peripheral nerve blocks and local anesthetics, are also less suitable in the setting of fast track recovery. Furthermore, in the case of local infiltration anaesthesia (LIA) –despite it being effective in controlling pain on the day of surgery– it is not effective in management of movement-evoked pain in the days following surgery.

Hence, driven by the need to overcome the limits to early rehabilitation posed by LIA and abductor canal block in the fast track protocol, our department decided to implement a relatively new patient-controlled analgesia system Zalviso® (AcelRx Pharmaceuticals, Redwood City, CA), approved for marketing in Europe in 2015. Zalviso is a novel drug/device delivery system for in-hospital management of moderate-to-severe post surgical pain. It provides patients with single-dose 15 μg sufentanil nanotablets with a 20-min lockout period. Upon need, the patient can access a sublingual nanotablet by pressing the handheld ZALVISO® dispenser, which is pre-programmed by the manufacturer and thus cannot be altered by users (nor hospital staff or patients), and which does not require the patient being connected to any IV infusion pumps (Sacerdote et al., 2016; Minkowitz et al., 2013).

Existing data from literature appear encouraging. A phase II study by Minkowitz et al. on the sublingual formulation in patients undergoing major surgeries, established 15 μg to be the optimal dosage in terms of efficacy and safety compared to lower dosages (superior analgesia and similar side effect profiles) (Minkowitz et al., 2013). Phase III studies have suggested analgesia to be superior compared to placebo (Jove et al., 2015; Melson et al., 2014; Busse et al., 2015) and equal to (but more rapid than) morphine administered through traditional IV-patient controlled analgesia (PCA) (Melson et al., 2014).

Among such studies, none however specifically address fast-track patient management in TKA. Therefore, our aim was to provide a pilot evaluation for such setting, comparing our standard of care (cFNB with multimodal analgesic approach) to the new SSTS Zalviso®.

## Methods

This was a retrospective observational study on a representative group of typical TKA hospital patients undergoing unilateral total knee replacement under perioperative multimodal analgesia, including data from patients consecutively admitted to the Hip and Prosthetic Orthopaedics Department, Humanitas Research Hospital in Rozzano (Milan, Italy) between July 2016 and May 2017. Specifically, the study compared a group of TKA patients receiving the new SSTS postoperative analgesia (SSTS group) versus a group of patients with matching characteristics who were treated according to our standard pain-management protocol by multimodal analgesia, i.e. cFNB, plus administration of oxycodone/naloxone, and ketoprofene (cFNB group/control).

Inclusion criteria were limited to age > 18 years, suitability for a fast track approach, and the patient’s ability to describe and report pain; whereas exclusion criteria were the administration of general anaesthesia during surgery, admission to the intensive care unit, history of chronic pain other than preoperative knee pain, alcohol or drug addiction, and cognitive or psychiatric disorders. All data used within the study were obtained through the patients’ hospital records and included patient demographic data, all data regarding intraoperative management and postoperative analgesia, and pain scores.

### Peri-operatory pain management

In accordance with our hospital protocol, general anaesthesia was not foreseen for this procedure. Both groups received sub-aracnoid anesthesia with bupivacaine 12/15 mg prior to surgery and LIA with ropivacaine 400 mg around the surgical wound immediately after surgery. In addition to these, the routine surgical protocol also included administration of ondansetron 4 mg, metoclopramide 10 mg, tranexamic acid 1000 mg in 200 ml saline (performed by the surgeon prior to the end of the surgical intervention), methylprednisolone 125 mg, and ranitinidine 50 mg.

After surgery, patients in the SSTS group received sublingual sufentanil SSTS (15 μg upon need) plus etoricoxib 120 mg (1 capsule/24 h) or –in the case of intolerance to FANS– paracetamol 1 g three times a day. Conversely, patients treated according to our standard protocol (cFNB group/control) underwent continuous femoral nerve block (cFNB) at the inguinal canal with continuous infusion of local anaesthetic (ropivacaine 0.125% 5 ml\h) and oxycodone/nalaxone10 mg/5 mg tablets twice daily plus ketoprofene 100 mg 2 capsule/24 h or, in the case of intolerance to FANS, paracetamol 1 g three times a day,

In the event pain was not properly controlled (NRS ≥4) by prescribed analgesic therapy, patients of both groups received a “rescue dose” of oral morphine 10 mg, upon need.

### Objectives, measurements and endpoints

The primary objectives were to (i) assess the efficacy of SSTS in fast track recovery (defined as NRS < 3 and ability to walk the same day of the procedure), and (ii) safety in comparison to cFNB. Secondary objectives of the study were to gather feedback on the ease of use of the Zalviso® drug delivery device system by healthcare staff and patients.

Endpoints measured were pain intensity at rest and upon movement, ability to stand and walk, number of discontinued treatment, number of side effects/adverse events, number of rescue doses length of hospital stay and usability scores. Specifically, pain was measured using the NRS numeric rating scale (range 0 = no pain, to 10 = worst pain possible). Movement-evoked NRS (mNRS) was also recorded in response to passive motion and in active transition to sitting and standing positions, and in motion. Pain scores available in patient records for both groups were the mean scores for T1 throughout T3 (T1 to T3 are the 3 days post surgery). For the SSTS group, further evaluations were recorded for the day prior to surgery (T-1), the day of surgery at delivery of the SSTS device to the patient (T0), the first administration of sufentanil sublingual nanotablets, and after 2, 4, 8 and 16 h from the first nanotablet.

The assessment on usability of the drug delivery device by staff and by patient was assessed through semi-quantitative data including time for preparation, number of doses administered and subjective evaluation scales (very difficult, difficult, normal, easy, very easy) measuring the nurse’s ease of use of the device, as well as the patients’ ability to understand the functioning of device and of putting instructions into practice.

### Ethical considerations

In accordance with our hospital regulation, the study was submitted to and evaluated by the internal Ethics Committee (Comitato Etico Indipendente – IRCCS Istituto Clinico Humanitas). As to informed consent, all patients being referred to our Institution signed a written informed consent for data analysis and result reporting upon admission.

### Statistical analysis

All data are reported as descriptive variables, normal distributed data should be displayed as means ± standard deviations (SD) and non-parametric data as median (interquartile range, IQR). Accordingly, to compare distributions between groups, we used T-test, non-parametric Kruskal-Wallis test or chi-square test. Pairwise post-hoc comparisons were performed with unpaired-samples t-test, Mann-Whitney test or chi-square test, as appropriate; in all cases we used a Sidak correction. Chi-square test was computed with Yates’ continuity correction for 2 × 2 contingency tables.

Pain intensity was evaluated through analysis of variance (ANOVA) for repeated measures with multiple comparisons versus the baseline. The baseline value is the average between the value at initial dose and any following (maximum 4) provided during the first 24 h and, for each visit (V1 on day 1, V2 on day 2, V3 on day 3), the average pain intensity between the four (at 7:00, 12:00, 18:00 and 21:00). Pain intensity between the groups was also evaluated using the Kruskal-Wallis test comparing the baseline observation with the most recent available data.

Statistical significance was set as *p* <  0.05 (5%). Data were analysed using SAS 9.4 SAS Institute Inc. 100 SAS Campus Drive Cary, NC 27513–2414, USA.

## Results

The study evaluated a total of 95 patients using the SSTS drug/device Zalvisio® (SSTS group) and compared it to a control group of 87 patients on continuous femoral nerve block (cFNB group) which was representative of the typical TKA hospital population, presenting similar characteristics as to age, comorbidities, type of operation with the SSTS group, differing limitedly to the analgesic technique received (Table 1). There was a statistical significance between groups for age and number of patients with hypertension, which, however, was not relevant from a clinical point of view.

**Table 1:** Demographics and clinical data of the two study groups

Characteristic	SSTS	cFNB	*p value*
	(95)	(87)	
Patient age (years)	66.2 ± 7.6	68.7 ± 6.6	< 0.05^a^
Female/Male	66/29	46/41	< 0.001
BMI (mean ± SD)	29.3 ± 5.0	29.8 ± 5.4	ns^a^
Comorbidities, n (%)			
Hypertension	63 (66.3)	36 (41.4)	< 0.001^a^
Diabetes	12 (12.6)	14 (16.1)	ns^b^
Heart diseases	13 (13.7)	12 (13.8)	ns^b^
Other	57 (60.0)	6 (6.9)	< 0.001^b^
Pain, mean ± SD			
NRS T1	1.96 ± 0.93	0.60 ± 1.06	< 0.001^b^
NSR T2	1.79 ± 0.90	0.40 ± 0.58	< 0.001^b^
NSR T3	1.21 ± 0.94	0.18 ± 0.42	< 0.001^b^
NRS T4	x	0.16 ± 0.41	
mNRS T1	1.85 ± 0.96	2.06 ± 1.9	< 0.001^b^
mNRS T2	1.62 ± 1.15	1.94 ± 1.5	< 0.01^b^
mNRS T3	1.16 ± 0.73	1.33 ± 0.93	< 0.05^b^
mNRS T4	x	1.94 ± 1.08	
Rescue analgesic, n (%)			
T1	5 (5.3)	22 (25.3)	< 0.001^c^
T2	0 (0.0)	13 (14.9)	
T3	0 (0.0)	9 (10.3)	
AEs, n (%)			
Overall n. events	7	132	< 0.001^c^
Overall n. patients (%) Nausea	1 (1)	42 (48)	< 0.001^c^
Vomiting	2 (1)	24 (28)	< 0.001^c^
PONV	2 (2)	61 (70)	< 0.001^3^
Erythema	1 (1)	5 (6)	ns
Dizziness	1 (1)	15 (17)	< 0.001^c^
Numbness	0	47 (54)	
LOS, n patients (%)			
= 4 days	95 (100.0%)	32 (36.8%)	
5–7 days	0 (0.0%)	55 (63.2%)	

*Abbreviations*: *AEs* adverse events, *BMI* body mass index, *LOS* length of hospital stay, *NRS* numeric rating score pain at rest, *mNRS* movement-evoked NRS. T1-T4, postoperative days 1–4

Statistical analyses used: ^a^T-Test; ^b^ Kruskal-Wallis; ^c^ Chi-squared test

Overall, patients from the SSTS group had significantly fewer adverse effects (with a total of 7 events experienced by 6% of patients vs 132 AEs experienced by 74% of cFNB patients), a slightly better pain control upon movement, and lesser need for supplemental rescue dose (5% vs 60%) compared to the cFNB group, which altogether allowed patients in the SSTS group to ambulate earlier after surgery and achieve the goals set by the fast track protocol. Moreover, patients from the SSTS group were all dismissed from the hospital within the three days from the intervention (LOS = 4 days) in keeping with the fast track protocol, compared to only 36% of the cFNB patients with the remaining having a median LOS (median, irq) of 5(2).

### Pain scores, adverse events, and ambulation

**Fig. 1:**
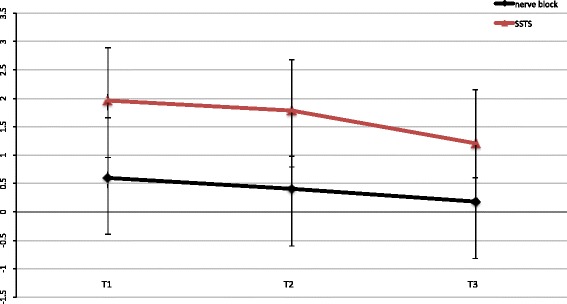
NRS for SSTS and cFNB. Pain scores at rest were lower in the cFNB group at all times T1-T3 (*p* < 0.05). (T1: day 1, T2: day 2, T3: day 3)

**Fig. 2:**
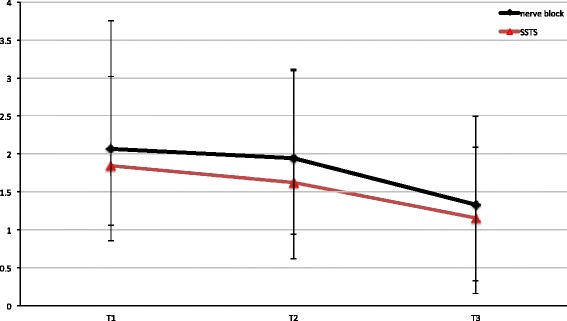
mNRS for SSTS and cFNB. Movement-evoked pain scores were lower in the SSTS group (*p* < 0.05). (T1:day 1, T2: day 2, T3: day 3)

**Fig. 3:**
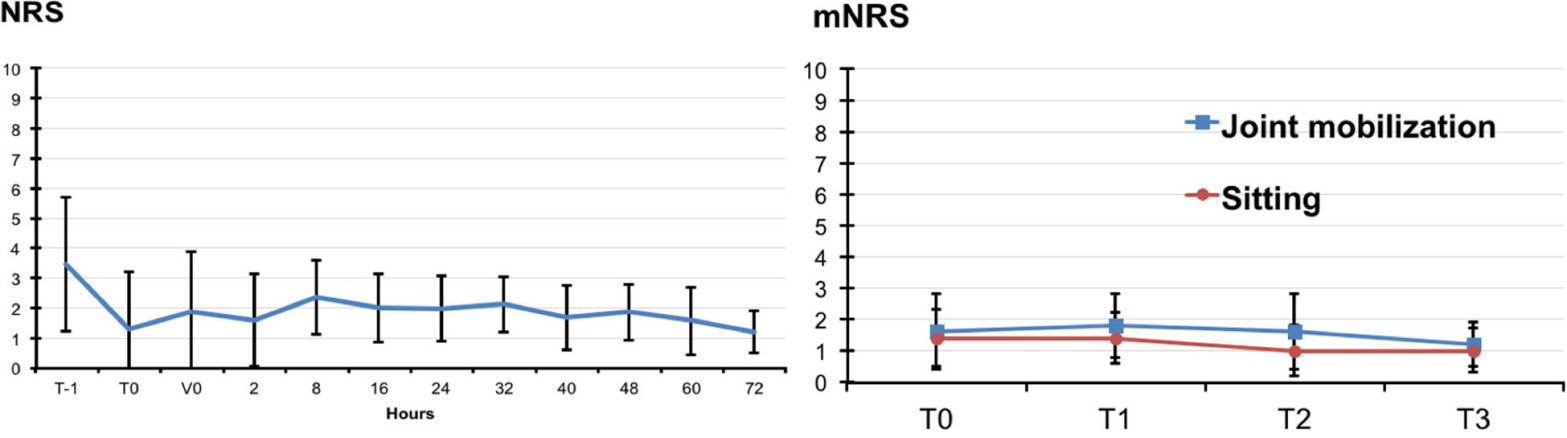
Pain scores in the SSTS group. Left panel: Pain intensity at rest (NRS) mean ± SD throughout the perioperative period. T-1: preoperative evaluation, T0: day of surgery, SSTS given to patient, V0: first nanotablet administration. Right Panel: movement-evoked pain (mNRS) mean ± SD from the day of surgery to the 3rd day after surgery

With specific reference to pain perception during the postoperative period T1 to T3, the cFNB group (control) had lower NRS at rest (*p* < 0.05) throughout T1- T3 compared to SSTS (Fig. 1), whereas movement-evoked pain scores, mNRS, were lower in the SSTS group at all time points measured (Fig. 2). The overall differences in pain intensity as well as difference in time points T1-T3 between groups were slight – despite statistical significance (*p* < 0.05) (Table 1). All patients (100%) of the SSTS group were able to stand and walk for 10 m from T1 on, whereas the patients in the cFNB group showed a slower recovery, with only 40% able to stand and walk on T1, 70% on T2 and 85% on T3. As a matter of fact, patients of the SSTS who were operated early in the day were actually able to ambulate already on the day of surgery T0. Figure 3 describes the NRS trend for the SSTS group. The comparison between baseline (T0) and our last data available showed a mean reduction of 37% (from a score of 1.9 to 1.2) in pain intensity, which was statistically significant (*P* < 0.01) as evidenced by the paired t-test.

The mean duration of treatment with SSTS (expressed as the time difference from the first to the last nanotablet received) was slightly less than 48 h, with no patients using SSTS for more than 72 h. On T0, the mean time from receiving the device and self-administration between tablets was 1.5 h to the first tablet, 5 h between the first and the second tablet, 3.5 h between the second and third tablet, and 2.5 for the fourth tablet. The mean consumption of tablets was 9 tablets during T0 (the day of surgery), 6 during T1, and 3 during T2, with all patients (97%), using less than (or at most) one cartridge (40 sufentanil nanotablets) throughout the entire postoperative period. While most patients were able to discontinue treatment early (before 72 h) given the absence of moderate-severe pain, 2 patients (2.1%) discontinued SSTS due to ineffective pain relief, and 2 (2.1%) due to the malfunctioning of the device.

Rescue doses were required less often in the SSTS group (5% of SSTS patients) than the cFNB control (60% patients, of which 25% on T1, 15% on T2 and 10% on T3 and 10% on T4).

Among the adverse effects reported were post-operative nausea and vomiting (PONV), erythema, numbness, and dizziness. The number of AEs was significantly lower (*p* < 0.001) in the SSTS group, with 7 events (7.4% of patients) in the SSTS group, compared to 132 events (73.6%) in the cFNB group (nausea 42%, numbness 34%, dizziness 23%, vomiting 17%, and PONV 14%) as shown Table 1. Also, a large percentage of patients in the cFNB group experienced several episodes of femoral catheter dislodgement (5.7% on T1, 14.9% on T2 and 25.3% on T3). In most cases, the occurrence of adverse effects and inconvenience with catheter cFNB administration required change or adjustment of treatment and extra time out of rehabilitation.

### Usability

The SSTS drug/delivery system in our population was typically prepared by a nurse, with a mean set up time 2.7 min (±1.8) (Table 2). Most patients confirmed that SSTS was easy to use, and reported no difficulty in understanding how to use the device (Table 3). Most of the patients reported they were not impaired in their movements, and 74% of patients were completely satisfied in terms of pain relief .

**Table 2:** Data on set up and use of the SSTS

Professional who delivered device to patient (n; %)	
Nurse	93 (97.9)
Anaesthesiologist	2 (2.1)
Time for SSTS set up - minutes (median, IQR)	2.4 (1.3–3.9)
Treatment duration - hours (mean, ±SD)	48 ± 19
Administered doses per patient (mean, ±SD)	17.9 ± 10.4
0–24 h	9.3 ± 5.9
24–48 h	5.7 ± 4.4
48–72 h	2.9 ± 3.7

**Table 3:** Patient evaluation on usability (n; %)

Score	Patient’s Understanding	Correct use by patient	Administration	Mobility
Very Difficult	0 (0)	0 (0)	0 (0)	0 (0)
Difficult	2 (2.1)	0 (0)	2 (2.1)	0 (0)
Normal	7 (7.4)	4 (4.2)	2 (2.1)	1 (1.1)
Easy	76 (80)	76 (80)	75 (78.9)	75 (78.9)
Very easy	8 (8.4)	11 (11.6)	12 (12.6)	14 (14.7)
unknown	2 (2.1)	4 (4.2)	4 (4.2)	5 (5.3)

## Discussion

The present study evaluated the efficacy and safety of Zalviso® sublingual sufentanil tablet system (SSTS) in comparison to our standard of care for postoperative pain management, a multimodal analgesic approach with cFNB, in a population of TKA patients managed according to fast track rehabilitation principles. Results from the study confirmed the efficacy and safety of the SSTS in TKA –in agreement with previous studies in literature– providing herein new evidence specifically in the fast track setting (Minkowitz et al., 2013; Jove et al., 2015; Melson et al., 2014).

Based on our experience, the most interesting advantage from the use of SSTS in the fast track setting was the lower incidence of adverse effects (AEs), compared to the cFNB group (7 events in the SSTS vs 132 in the cFNB) and the satisfactory control of pain which determined the patients’ (i) ability to comply with the fast track rehabilitation program and (ii) their fitness for dismissal on T3, the third day after surgery.

### Efficacy, safety, and ambulation

Overall, the adverse effects were significantly fewer in the SSTS group compared to the cFNB group. The larger number of AEs experienced by patients in the cFNB group appeared most likely to be due to the higher rate of adjunctive analgesic “rescue doses” (in addition to oxycodone/naloxone as by standard protocol) needed to compensate for the scarce management of pain, and thus to the higher exposure to opioids. Certainly, this was to some extent linked to analgesic gaps and adjustments to the analgesic protocol ensuing from catheter dislodgement.

Another key factor in the better outcome for patients receiving SSTS was also the good control over pain. Patients using Zalviso® (SSTS group) reported lower pain upon movement than cFNB patients (control group) at all times measured, and pain was properly managed also when LIA had worn off. Despite the difference in pain scores between the two groups was minimal in terms of absolute values, these were, however, clinically relevant as well as meaningful to the patient in terms of ambulation and function. As suggested by several authors, evaluations derived from patient reported outcomes on pain should be considered within a broader concept of “minimally important differences” and of “thresholds of improvement” and should be considered when evaluating success of interventions (Busse et al., 2015).

The good control over pain was further suggested by the difference in rate of rescue doses, which is indicative of the efficacy of the treatment adopted (i.e., the better pain management, the fewer the rescue doses). In fact, rescue dose was needed for 60% of patients on cFNB compared to only 5% of those using SSTS.

It is noteworthy to mention here sufentanil’s pharmacodynamics and pharmacokinetic properties and the regulated release of the sublingual formulation that contribute to Zalviso’s effectiveness. Compared to morphine IV PCA, sufentanil features high liposolubility and fast equilibration between plasma and central nervous system (t_1/2_K_e0_ of 6 min compared to 168 min with morphine), and its sublingual administration provides rapid onset of analgesic activity with a constant median plasma half-time following repeated administration (Sacerdote et al., 2016). Importantly, sufentanil displays a higher therapeutic index (26.000 vs 71 with morphine) and lacks active metabolites (Babazade & Turan, 2016), thus allowing a quicker achievement of optimal analgesia without the risk of accumulation and major side effects. A 15 μg dose of sublingual sufentanil is equivalent to 3–4 mg of IV morphine (Melson et al., 2014; Babazade & Turan, 2016).

The optimal pharmacokinetic profile and clinical features of SSTS in the treatment of acute postoperative pain have already been confirmed in recent phase II and III studies, in which the SSTS was administered as single treatment, and indeed our results are in agreement with data in literature (Minkowitz et al., 2013; Jove et al., 2015; Melson et al., 2014; Ringold et al., 2015).

As to the type of AEs, the most frequently occurring were nausea and vomit, despite prophylaxis against PONV had been prescribed in both groups. Events of PONV were fewer and with lower incidence in the SSTS group. There were no reports of major complications such as respiratory depression or cognitive dysfunction in either group. Pruritus, which was evidenced as a possible side effect with sufentanil (randomized trial by Minkowitz et al. comparing efficacy of SSTS in several dosing arms and against placebo, across a broad range of general surgical scenarios other than TKA) (Minkowitz et al., 2013) was reported in the SSTS group for one patient only.

### Usability

Finally, as to usability, SSTS was well accepted by both patients and care providers. Nurses described its set up as easy and quick (approximately 4 min per patient) and its functioning easy to explain to patients. While these aspects may appear trivial, from an operational point of view they translate in saving of time, easier streamlining of procedures, and better prevention of human error (Turnbull et al., 2017).

As evidenced in a recent review by Schein et al. on PCA medication errors, at least 4.5% of patients in the US each year are affected by IV PCA errors linked to medical prescriptions, drug choice/combinations, dose titrations, pump programming, and modifiable operator/patient dosing of analgesic opioid (Schein et al., 2009; Hicks et al., 2008). In our patient population the use of Zalviso® contributed to reduce such risks altogether, by eliminating possible sources of operator or patient manipulation —thanks to the single-dosed nanotablets and pre-programmed lockout interval set by the manufacturer which did not require any further operator-dosing interventions. Moreover, the device’s programmed lockout period system also eliminates the risk of opioid overdose by preventing patients from accessing tablets too close in time from one another, while the sublingual formulation maximized absorption and reduced the risk for analgesic gaps. One last advantage of Zalviso® with sublingual tablets is that it avoided the need for the physical connection to a PCA pump or IV pole, which generally reduce mobility and involve the classic risks of infection, analgesic gaps due to IV catheter infiltration or IV tubing obstruction (Panchal et al., 2007; Palmer & Miller, 2010).

Patients mostly complied with the drug/device instructions, and reported tablet administration as “easy” or “very easy”. This is also an aspect that should not be underestimated, if one considers that IV PCAs generally require more training nursing staff and more nurse-patients interaction time (e.g. patient calling more frequently for reassurance on correct functioning of IV administration, questions or issues with catheter) (Sacerdote et al., 2016). Moreover, patients appreciated the fact of being an active part in their treatment. Not depending on others made them more aware and motivated in adhering to the rehabilitation program.

### Suitability of SSTS in fast track TKA

All considered, the most interesting aspects we can gather from these results with SSTS are (i) the effectiveness in managing movement-evoked pain which allowed the patient to ambulate right from T0 (i.e. same day of surgery) and the days after surgery, (ii) the reduction of negative stimuli (pain, opioid-related side effects, poor muscular engagement, physical connection to a infusion line, catheter dislodgment) that could interfere with the fast track rehabilitation, (iii) the high patient compliance to analgesic treatment, (iv) the simplicity of use for both health professionals and patients.

### Limits of the study

With no doubt, these results must be considered as evidence of real-life clinical practice, and cannot have the strength of a randomized controlled trial. Future targeted randomized studies could further evaluate SSTS by addressing parameters such as “minimally important difference” and “threshold of improvement”, as suggested recently by several working groups on patient reported outcomes on pain. Nonetheless we believe this study provides useful information on pain management in the fast track approach of TKA setting.

## Conclusion

In conclusion, results from this pilot study proved Zalviso® SSTS to be particularly suitable in the multimodal analgesic approach in a fast track protocol. The fewer adverse events and lower pain scores for the SSTS group were associated to a better ability to ambulate early after surgery compared to cFNB, which allowed patients to go through the fast track management and be dismissed on the third day after surgery.

From the point of view of principles of pain physiology, the high levels of efficacy for the management of dynamic pain represents somewhat an interesting outcome –particularly when compared to the other standards of pain management, such as regional continuous techniques.

## References

[CR1] Albrecht E, Guyen O, Jacot-Guillarmod A, Kirkham K (2016). The analgesic efficacy of local infiltration analgesia vs femoral nerve block after total knee arthroplasty: a systematic review and metaanalysis. BJA.

[CR2] Allegri M, Bugada D, Grossi P (2016). Italian registry of complications associated with regional anesthesia (RICALOR). An incidence analysis from a prospective clinical survey. Minerva Anestesiol.

[CR3] Babazade R, Turan A (2016). Sufentanil sublingual tablet system for the management of postoperative pain. Expert Opin Pharmacother.

[CR4] Bugada D, Allegri M, Gemma M, Ambrosoli AL, Gazzerro G, Chiumiento F, Dongu D, Nobili F, Fanelli G, Ferrua P, Berruto M, Cappelleri G (2017). The effects of anaesthesia and analgesia on long-term outcome after total knee replacement: a prospective, observational, multicentre study. Eur J Anaesthesiol.

[CR5] Busse J, Bartlett S, Dougados M et al (2015) Optimal Strategies for Reporting Pain in Clinical Trials and Systematic Reviews: Recommendations from an OMERACT 12 Workshop. J Rheumatol First Release May 15 2015. 10.3899/jrheum.141440 10.3899/jrheum.141440 25979719

[CR6] Carli F, Kehlet H, Baldini G (2011). Evidence basis for regional anesthesia in multidisciplinary fast-track surgical care pathways. Reg Anesth Pain Med.

[CR7] Chan EY, Fransen M, Parker DA, Assam PN, Chua N (2014) Femoral nerve blocks for acute postoperative pain after knee replacement surgery. Cochrane Database Syst Rev. 10.1002/14651858.CD009941.pub2 10.1002/14651858.CD009941.pub2 PMC7173746 24825360

[CR8] den Hertog A, Gliesche K, Timm J, Muhlbauer B, Zebrowski S (2012). Pathway-controlled fast-track rehabilitation after total knee arthroplasty: a randomized prospective clinical study evaluating the recovery pattern, drug consumption, and length of stay. Arch Orthop Trauma Surg.

[CR9] Elkassabany NM, Antosh S, Ahmed M (2016). The risk of falls after Total knee Arthroplasty with the use of a femoral nerve block versus an Adductor Canal block: a double-blinded randomized controlled study. Anesth Analg.

[CR10] Hicks RW, Sikirica V, Nelson W, Schein JR, Cousins DD (2008). Medication errors involving patient-controlled analgesia. Am J Health Syst Pharm.

[CR11] Ilfeld BM (2017). Continuous peripheral nerve blocks: an update of the published evidence and comparison with novel, alternative analgesic modalities. Anesth Analg.

[CR12] Jove M, Griffin DW, Minkowitz HS, Ben-David B, Evashenk MA, Palmer PP (2015). Sufentanil sublingual tablet system for the Management of Postoperative Pain after knee or hip Arthroplasty: a randomized, placebo-controlled study. Anesthesiology.

[CR13] Kehlet H, Thienpont E (2013). Fast-track knee arthroplasty -- status and future challenges. Knee.

[CR14] Lotsch J (2005). Pharmacokinetic-pharmacodynamic modeling of opioids. J Pain Symptom Manag.

[CR15] Melson TI, Boyer DL, Minkowitz HS (2014). Sufentanil sublingual tablet system vs. intravenous patient-controlled analgesia with morphine for postoperative pain control: a randomized, active-comparator trial pain practice: the official journal of world institute of. Pain.

[CR16] Minkowitz HS, Singla NK, Evashenk MA (2013). Pharmacokinetics of sublingual sufentanil tablets and efficacy and safety in the management of postoperative pain. Reg Anesth Pain Med.

[CR17] Mudumbai S, Kim T, Howard S (2014). Continuous adductor canal blocks are superior to continuous femoral nerve blocks in promoting early ambulation after TKA. Clin Orthop Relat Res.

[CR18] Palmer P, Miller R (2010). Current and developing methods of patient-controlled analgesia. Anesthesiol Clin.

[CR19] Panchal SJ, Damaraju CV, Nelson WW, Hewitt DJ, Schein JR (2007). System-related events and analgesic gaps during postoperative pain management with the fentanyl iontophoretic transdermal system and morphine intravenous patient-controlled analgesia. Anesth Analg.

[CR20] Pelt C, Anderson A, Anderson M, Van Dine C, Peters C (2014). Postoperative falls after Total knee Arthroplasty in patients with a femoral nerve catheter: can we reduce the incidence?. J Arthroplasty.

[CR21] Rawal N (2016). Current issues in postoperative pain management. Eur J Anaesthesiol.

[CR22] Ringold FG, Minkowitz HS, Gan TJ (2015). Sufentanil sublingual tablet system for the management of postoperative pain following open abdominal surgery: a randomized, placebo-controlled study. Reg Anesth Pain Med.

[CR23] Rodriguez-Merchan C (2015). Pros and cons of fast-track Total knee Arthroplasty. Int J Orthopaed.

[CR24] Sacerdote P, Coluzzi F, Fanelli A (2016). Sublingual sufentanil, a new opportunity for the improvement of postoperative pain management in Italy. Eur Rev Med Pharmacol Sci.

[CR25] Schein JR, Hicks RW, Nelson WW, Sikirica V, Doyle DJ (2009). Patient-controlled analgesia-related medication errors in the postoperative period: causes and prevention. Drug Saf.

[CR26] Turnbull Z, Sastow D, Giambrone G, Tedore T (2017). Anesthesia for the patient undergoing total knee replacement: current status and future prospects. Local and Regional Anesthesia.

